# Ovarian Follicular Growth through Intermittent Vaginal Gonadotropin Administration in Diminished Ovarian Reserve Women

**DOI:** 10.3390/pharmaceutics14040869

**Published:** 2022-04-15

**Authors:** Chao-Chin Hsu, Isabel Hsu, Li-Hsuan Lee, Rosie Hsu, Yuan-Shuo Hsueh, Chih-Ying Lin, Hui Hua Chang

**Affiliations:** 1 Taiwan United Birth-Promoting Experts Fertility Clinic, Tainan 710, Taiwan; 2 Department of Obstetrics and Gynecology, National Taiwan University Hospital, Taipei 104, Taiwan; izserx@gmail.com; 3 Department of Obstetrics and Gynecology, National Cheng Kung University Hospital, Tainan 701, Taiwan; 4 Nepean Hospital, Penrith, NSW 2750, Australia; lihsuan.lee@health.nsw.gov.au; 5 Department of Pediatrics, National Taiwan University Hospital, Taipei 104, Taiwan; raisin_isbest@hotmail.com; 6 Department of Medical Science Industries, College of Health Sciences, Chang Jung Christian University, Tainan 711, Taiwan; yshsueh@mail.cjcu.edu.tw; 7 Institute of Clinical Pharmacy and Pharmaceutical Sciences, College of Medicine, National Cheng Kung University, Tainan 701, Taiwan; ilg811280@gmail.com; 8 School of Pharmacy, College of Medicine, National Cheng Kung University, Tainan 701, Taiwan; 9 Department of Pharmacy, National Cheng Kung University Hospital, College of Medicine, National Cheng Kung University, Tainan 701, Taiwan; 10 Department of Pharmacy, National Cheng Kung University Hospital, Dou-Liou Branch, Yunlin 640, Taiwan

**Keywords:** gonadotropins, vaginal administration, poor ovarian response, diminished ovarian reserve, POSEIDON group, controlled ovarian hyperstimulation, in vitro fertilization

## Abstract

It is a challenge to obtain enough oocytes during in vitro fertilization (IVF) in women who have a poor ovarian response (POR) in achieving conception. We have adopted the characteristics of the first uterine pass effect, which we pioneered in employing the vaginal administration of gonadotropins in women receiving IVF treatments. In our previous study employing vaginal administration, faster absorption and slower elimination of gonadotropins were demonstrated, and, female subjects presented proper ovarian follicle growth and pregnancy rates. In this study, during 2016–2020, 300 to 675 IU of gonadotropins were administered vaginally every three days in 266 POR women for their controlled ovarian hyperstimulation (COH). The injections were performed with needles angled at 15–30° towards the middle-upper portions of the bilateral vaginal wall, with an injection depth of 1–2 mm. For the COH results, these women, on average, received 3.0 ± 0.9 vaginal injections and a total dose of 1318.4 ± 634.4 IU gonadotropins, resulting in 2.2 ± 1.9 mature oocytes and 1.0 ± 1.2 good embryos. Among these embryos, 0.9 ± 1.0 were transferred to reach a clinical pregnancy rate of 18.1% and a live birth rate of 16.7%. In conclusion, the intermittent vaginal administration of gonadotropins proved to be effective in POR women for their IVF treatments.

## 1. Introduction

The woman’s body presents a number of unique anatomical features, including the vagina, the endocervix, the uterus, and the ovary, all of which constitute valuable routes for the administration of drugs [1]. The vagina is a well-known route of drug delivery, particularly for the administration of hormones [2]. Despite being the most easily accessible female organ, the vagina has been overlooked as the route for drug administration by most gynecologists, including reproductive endocrinologists, in treating infertile women. Due to poor absorption across the vaginal epithelium, it has been a challenge to successfully deliver drugs through the vagina [3]. This situation has been improved in the past decade, and the vagina has become a potential route for the delivery of peptides and other macromolecules for therapeutic purposes [4,5,6].

A “first uterine pass effect” was suggested two decades ago, when steroid hormones were delivered through the vagina [7,8,9]. Delivery of progesterone supplements via the vagina compared to delivery of similar medication via the conventional intramuscular route showed up to 10× higher circulating levels of progesterone [10,11,12,13]. Blood circulation is a crucial mechanism of the “first uterine pass effect,” and the large surface area and blood supply of the vaginal epithelium allows for more efficient systemic drug delivery of peptides and other therapeutic macromolecules [3,14]. This mechanism is based on the countercurrent exchange via vein-to-artery diffusion of the uterine arteries in the upper vagina. These characteristics opened a wider door for the administration of medicine, with a special focus on the treatment of uterine disorders, as well as the enhancement of ovarian folliculogenesis.

We adopted these characteristics and pioneered the vaginal administration of gonadotropins (Gns) in women receiving in vitro fertilization (IVF) treatments. In our previous pilot study, proper folliculogenesis, fertilization rates, implantation rates, and pregnancy rates were demonstrated through vaginal injections of the recombinant human follicle stimulating hormone (rhFSH) in women receiving IVF treatment [15,16]. In the pharmacokinetic study, our results showed that female subjects receiving rhFSH through vaginal administration presented with faster absorption and slower elimination. Significantly lower times of maximum observed concentration (Tmax), clearances (CL), elimination rate constants (K10), higher areas under curve (AUC), and half-lives of elimination (T_1/2_ elimination) were noted, compared with those received through abdominal administration [17,18]. In the clinical parameters, we found that the female subjects receiving rhFSH using intermittent vaginal administration employing lower doses of rhFSH had similar ovarian follicle growth, mature oocytes, and pregnancy rates, compared to those who received conventional abdominal administration [17,18]. Furthermore, in an animal study, follicular growth and ovulation were induced more efficiently with lower frequencies of FSH injection through the vagina. Higher serum levels of progesterone and the expression of progesterone receptors in the local endometrium were also found [18].

Worldwide, infertility affects approximately 8–12% of couples of reproductive age, and many of them need assisted reproductive techniques to achieve conception [19]. Age-related infertility is steadily increasing worldwide, with many women presenting with a diminished ovarian reserve. The Bologna criteria were developed in 2011 to standardize the definition of poor ovarian response (POR) [20]. In women with a POR, COH yields a very small number of follicles [21]. Various treatment regimens have been designed to manage patients with POR, including high doses of Gns, natural and modified natural cycles, supplementation with luteinizing hormones (LHs), luteal antagonists and letrozole co-treatment, and growth hormones [21,22,23,24]. In spite of the variety of regimens, there is no consensus on an ideal COH protocol for patients identified as poor ovarian responders [25,26]. More recently, the POSEIDON (Patient-Oriented Strategies Encompassing IndividualizeD Oocyte Number) criteria were developed to define “low prognosis” women undergoing IVF treatments [27]. POSEIDON group 4 women—“patients > 35 years with poor ovarian reserve (antral follicle count (AFC) < 5, anti-Müllerian hormone (AMH) < 1.2 ng/mL)”—have higher amounts of aneuploid embryos and, in turn, an increased need for multiple IVF cycles, with significantly poorer prognoses.

In this study, we aimed to investigate a population of POR women, a majority of whom were POSEIDON group 4 women who received vaginal administration of Gns for their controlled ovarian hyperstimulation (COH) in their assisted conception treatments. Our results indicate the efficacy of this administration mode in POR women.

## 2. Materials and Methods

### 2.1. Study Population and Design

We approached women who had requested IVF treatments from January 2016 to December 2020. Only those POR women receiving IVF treatments were included as the study group. The inclusion criteria were POSEIDON groups of infertile women aged over 35 years with a body mass index (BMI) of 17.0–28.0 kg/m^2^. The exclusion criteria included history of allergic reactions, history of recurrent miscarriages, coagulation disorders, use of hormonal preparations in the last 3 menstrual cycles, and—last but not least—unexplained infertility. Women were categorized into three groups based on the initial serum AMH serum level: Group A women with impending ovarian failure (AMH < 0.5 ng/mL); Group B women with poor-to-diminished ovarian reserve (0.5 ≤ AMH < 1.2 ng/mL) (Groups A and B belonged to POSEIDON group 4); and Group C women (AMH ≥ 1.2 ng/mL) (POSEIDON group 2a).

### 2.2. Ethics Approval

This study was performed in accordance with the Declaration of Helsinki’s Good Clinical Practice standards, and with local regulatory requirements; it was approved by the Institutional Review Board (TSMH IRB/Protocol No: 18-115-B). All patients included in the study provided written consent and were treated at the IVF Unit at the TUBE Fertility Clinic, Tainan, Taiwan, under a license from the Taiwan Department of Health Authority. The potential adverse reactions, as well as the benefits and risks of vaginal medication administration, were advised. The possibility of local skin allergic reactions and the risk of ovarian hyperstimulation syndrome (OHSS) were fully explained.

### 2.3. Controlled Ovarian Hyperstimulation

The recruited participants proceeded primarily in accordance with the gonadotropin-releasing hormone-antagonist (GnRH-antagonist) protocol. Enrolled subjects first visited the IVF unit on menstrual cycle day 2, and ultrasound examinations were performed to rule out any ovarian pathologies before the administration of Gns. The injections of Gns were in accordance with our previously established method [17,18]. The injections were given with sterile single-use syringes and needles. Standard hygiene precautions, including the usage of gloves and sterile procedures, were followed for the prevention of local infection and the avoidance of contamination of materials during the medication administration process.

**Figure 1:**
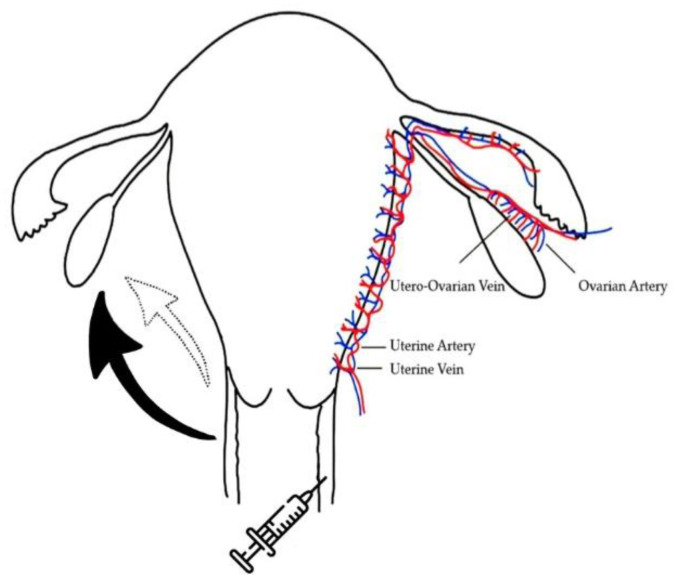
Schematic expression of the vaginal administration of gonadotropins and the possible transportation routes to the ovary. The black solid arrow indicates the major transportation of medicines through lymphatic and venous circulations. The blank dotted line arrow indicates minor transportation of medicines via the countercurrent exchange through vein-to-artery diffusion or direct diffusion.

During the procedure, the patients were first placed in the Trendelenburg position. Then, ultrasonographic scanning was performed, followed by the insertion of a sterilized speculum for thorough cleansing of vaginal discharge with sterilized normal saline. Gonadotropin 300 IU (Gonal-f Prefilled Pen rhFSH in 0.5 mL, Merck Serono S.p.A., Modugno, Italy) in combination with 375 IU menopur (menotrophin; 75 IU FSH and 75 IU LH; Ferring GmBH, Kiel, Germany) was initiated on day 2 of the IVF cycle. The gonadotropin was aspirated into a 1 mL syringe fitted with a 30 gauge × 1/2 inch (0.31 × 13 mm) needle. The rhFSH was injected into the middle-to-upper portion of the vagina, at a depth of 1–2 mm [16,17] at approximately the 3 and 9 o’clock positions (Figure 1), with the needle angled at 15–30° toward the vaginal mucosa. Abstinence was requested for the avoidance of interference with the vaginal blood flow. Otherwise, the patients were allowed to return to normal daily activities after the procedure.

**Figure 2:**
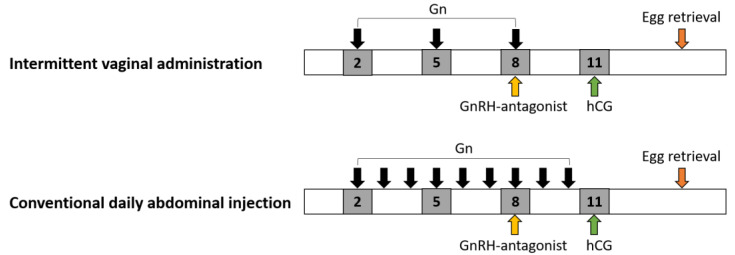
Schematic expression of gonadotropin (Gn) administration. The Gn was given on days 2, 5, and 8 for intermittent vaginal administration, in comparison to conventional abdominal injections that are performed daily starting on menstrual day 2. GnRH (Gn-releasing hormone) antagonist was given when the leading follicle reached 12–14 mm in diameter, which was mostly noted at day 8 of the cycle. hCG: human chorionic gonadotropin.

For all subjects, ultrasound scanning and serum hormone levels were recorded on menstrual cycle days 2, 5, 8, and 11, in accordance with the day for successive intermittent Gn injection and, in some women, until follicle maturation (Figure 2). On days 5 and 8, if follicular growth failed to meet the criteria for egg retrieval, with less than 1 follicle ≥ 15 mm (1 or more follicles ≥ 15 mm) for egg retrieval, then a second or third dose (300 to 675 IU) of Gn injection (300 to 675 IU) was administered, employing the same administration mode. GnRH antagonist (Orgalutran 0.25 mg, Vetter Pharma-Fertigung GmbH & Co., KG, Ravensburg, Germany) was initiated when the leading follicle reached 12 Vet mm in diameter. 2D sonographic scans with a 5.0-megahertz transvaginal transducer were performed by the same observer (C.C. Hsu) to track follicular growth by calculating follicle diameter (the mean diameter measured in two dimensions). The uterine endometrium thickness and bilateral uterine artery blood perfusion, including the pulsatility index and the resistance index, were measured regularly via color Doppler flow examination on the day of human chorionic gonadotropin (hCG) injection.

### 2.4. Oocyte Retrieval and Clinical Outcomes

The oocyte retrieval, in vitro fertilization, and cryopreservation procedures were performed in accordance with our previously established method. In brief, the final follicle maturation was triggered using 6500 IU recombinant hCG (Ovidriel, 250 micrograms, choriogonadotropin alfa, equivalent to approximately 6500 IU hCG. Merck Serono S.p.A., Modugno, Italy); when one or more follicles reached ≥15 mm in diameter, oocyte retrieval was performed 36 h later. The mature oocytes obtained were fertilized by intracytoplasmic sperm injection (ICSI) or cryopreserved. The immature oocytes were cultured for 24 h and then fertilized, if mature. The Kitazato cryotech vitrification method was employed, with a maximum of two oocytes per cryotop (VT 601; KITAZATO Shizouka 416-0907, Japan). A continuous single culture medium (NX complete CSCM-NXC with gentamicin and HAS; FUJIFILM Irvine Scientific, Santa Ana, CA, USA) was used for insemination and embryo culture. ICSI was performed 4–5 h after oocyte retrieval. The presence of two pronuclei was observed 16 h after ICSI. The Istanbul consensus [28] was used for the grading and evaluation of the growth of embryos at 68 ± 1 h after ICSI. Most day-3 cleavage-stage embryos were transferred back to the uterus in the fresh cycle under the guidance of transabdominal sonography. No more than two embryos were transferred if the patient was 35–40 years old, and no more than three embryos were transferred if the patient was ≥40 years old. The surplus embryos were cryopreserved mostly at the day-3 cleavage stage or cultured to the blastocyst stage. Luteal phase progesterone support, using micronized progesterone (utrogesterone, Besins Healthcare, Ayutthaya, Thailand) 100 mg three times daily, was started on the day after oocyte retrieval, and continued until 10 weeks of the gestation. Ultrasound scanning was performed 4 weeks after embryo transfer to identify the presence of clinical pregnancy. The adverse events and safety endpoints included recording the proportion of women with moderate-to-severe OHSS, as well as pain or other reactions.

### 2.5. Study Outcome Measures

The primary outcomes included the clinical parameters, such as the total dosage of Gns used and the number of Gn injections, the targeted ovarian response (follicular growth and mature oocytes retrieved), and the serum FSH and E2 concentrations. The secondary outcomes included the embryologic parameters, such as fertilization rates and embryonic growth, as well as clinical pregnancies and live birth rates on the basis of per oocyte retrieval. The ovarian sensitivity index (OSI: the dose of rhFSH used divided by number of mature oocytes obtained) [29], the follicular output rate (FORT: the ratio of pre-ovulatory follicle count (14–22 mm in diameter) on hCG day × 100/small antral follicle count (3–8 mm in diameter) at baseline) [30], and the follicle-to-oocyte index (FOI: the ratio between the number of oocytes obtained and the number of antral follicles at the beginning of COS) [31] were analyzed to determine the dynamic aspects of the follicular response to COS.

### 2.6. Measurement of Serum Hormone Levels

The Beckman Coulter ACCESS immunoassay system was applied in the hormone assay (UniCelDxl 800, Beckman Coulter, Brea, CA, USA). A sequential two-step immunoenzymatic “sandwich” assay was used to measure FSH and luteinizing hormone (LH) serum levels. For both FSH and LH, the assay could detect the lowest level of 0.2 IU/L, with a total imprecision of ≤10%. On the other hand, a simultaneous 1-step immunoenzymatic (“sandwich”) assay was used to measure anti-Müllerian hormone (AMH) serum levels. The 1-step assay has an upper limit detection of ≤0.02 ng/mL and a total imprecision of ≤10.0% at concentrations of ≥0.16 ng/mL. Last but not least, the competitive binding immunoenzymatic assay was used for the analysis of estradiol and progesterone serum levels, with the lowest detectable levels of 20 pg/mL for estradiol and 0.10 ng/mL for progesterone.

### 2.7. Statistical Analysis

Statistical analysis was performed using the Statistical Package for Social Sciences 23.0 (SPSS Inc., Chicago, IL, USA). Categorical variables were expressed as numbers and percentages and assessed using chi-square tests. Continuous variables were expressed as the means ± standard deviations (SD) and assessed using one-way ANOVA followed by Bonferroni post hoc tests. The level of significance was set at 0.05 for two-sided tests.

## 3. Results

### 3.1. Demographic of the Participants

**Figure 3:**
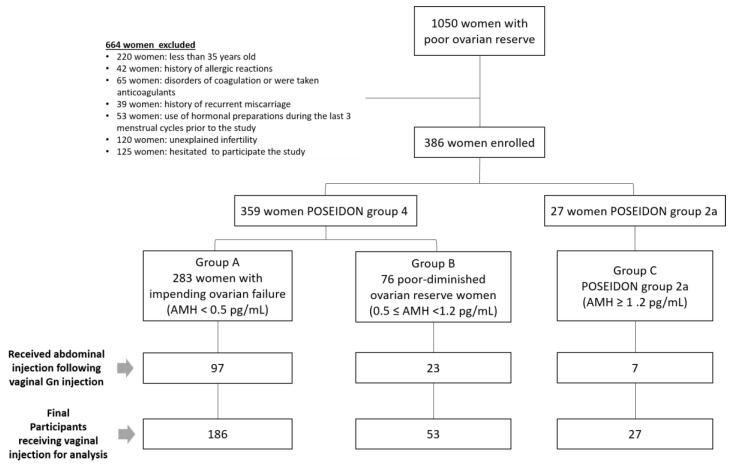
Flowchart for women of poor ovarian reserve receiving treatment.

There were 1886 women undergoing IVF treatments in 2016–2020 at our unit, and 1050 women who belonged to a POSEIDON group were approached for this study. Among these women, 220 who were less than 35 years old, 42 who had a history of allergic reactions, 65 who had coagulation disorders or were taking anticoagulants, 39 who had a history of recurrent miscarriage, 120 who had unexplained infertility, and 53 who had received hormonal preparations within the last three menstrual cycles were excluded. Another 125 women who hesitated to receive vaginal administration were also excluded. In the end, 386 women were enrolled to receive the vaginal Gn administration. Among these 386 women, 120 who received abdominal Gn injections following the first or second dose of vaginal administration were excluded from this study. All in all, a total of 266 women participated and received vaginal administration of Gns for their COS (Figure 3). The demographic characteristics of the subjects are shown in Table 1. The average age of the participants was 40.3 ± 4.2 years-old, with an average BMI of 22.1 ± 3.0 kg/m^2^. Their average duration of infertility was 5.5 ± 4.6 years (Table 1). Primary infertility was found in 118 women (44.5%). Many of the women had diminished ovarian reserves with low AMH serum levels. The average AFC was 2.8 ± 2.2. The causes of infertility included the following: ovulatory dysfunction in 147 cases (55.5%); other ovulatory problems in 104 women (39.2%); endometriosis and/or adenomyosis for 63 women (23.8%); tubal factors in 22 women (8.3%); and male factors in 15 cases (5.7%).

### 3.2. Clinical Patterns after Vaginal Administration of Gns

For COS, the participating women received 3.0 ± 0.9 vaginal Gn injections with a total Gn dose of 1318.4 ± 634.4 IU. The average duration of Gn exposure was 9 days, with the day of hCG at 10.4 days. The women were categorized into three Groups (A–C) based on the serum AMH levels. Women with impending ovarian failure (Group A, *n* = 185, AMH 0.16 ± 0.15 ng/mL) received an average of 1343.1 IU Gns. Women with poor-to-diminished ovarian reserves (Group B, *n* = 53, AMH 0.72 ± 0.18 ng/mL) received an average of 1291.9 IU of Gn. Women in Group C (*n* = 27, AMH 2.55 ± 1.31 ng/mL) received 1191.6 IU of Gns on average. Thus, all of the women received, on average, three doses of vaginal injection to complete their COS processes (Table 2).

**Figure 4:**
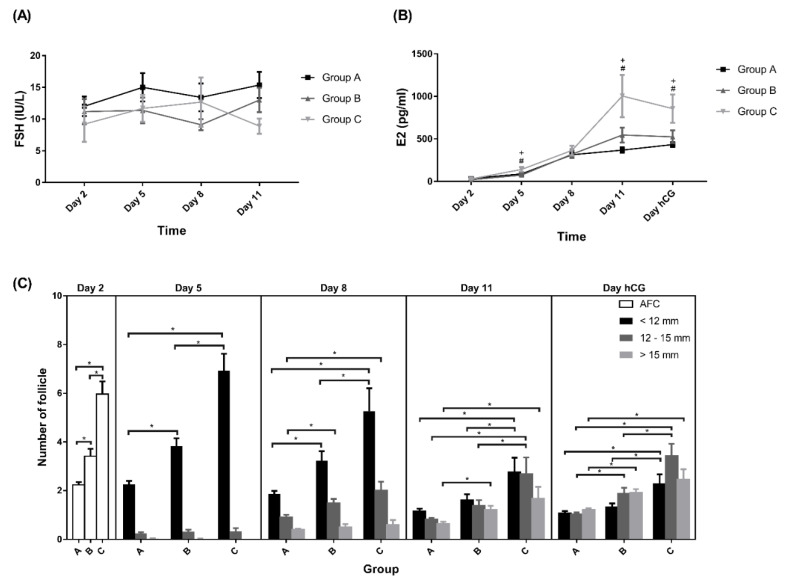
Levels of (**A**) FSH, (**B**) E2, and (**C**) follicles. All data presented as mean ± SEM. ^+^ The *p*-value difference was significant between Group C and Group A. ^#^ The *p*-value difference was significant between Group B and Group A. * *p* < 0.05. Group A: AMH < 0.5 ng/mL; Group B: 0.5 ng/mL ≤ AMH < 1.2 ng/mL; Group C: AMH ≥ 1.2 ng/mL.

Elevated serum FSH concentrations surpassing the FSH threshold levels (5–8 IU/mL) [32] for ovarian follicular growth were recorded in almost all subjects on days 5, 8, and 11 after vaginal administration of the Gns, and no differences in serum FSH level was observed among the three groups (Figure 4A). Only two participants with serum FSH levels less than 5 IU (<0.01%) were detected throughout the whole COH process.

The serum E2 levels were not different between days 1 and 8 of COS. However, higher levels were observed on days 5, 11, and the day for hCG triggering in Group C (Figure 4B). Reduced serum estradiol levels before the hCG-triggering day were noted in 16.30%, 24.5%, and 25.9% of subjects in Groups A, B, andC, respectively.

There were higher serum LH levels at baseline and on the day for hCG in Group A. While the percentage of women with elevated LH over 15 IU/L was also higher in Group A, an elevated serum LH (>15 IU/L) was noted in 24.8%, 5.1%, and 0% of the subjects in in Groups A, B, and C, respectively.

A premature progesterone rise (>1.5 ng/mL) before hCG-triggering was noted in 26.1% of the women, which was 25.8%, 20.5%, and 37.5% in in Groups A, B, and C, respectively.

### 3.3. IVF and Follicular Response after Vaginal Administration of Gns

Regarding follicle growth, there was no significant difference in the size of medium and large follicles on day 5 among the three groups. However, significantly higher numbers of small, medium, and large follicle were noted in Group C on days 8, 11, and the day of hCG (Figure 4C). The distribution of numbers of small, medium, and large follicles on the day of hCG, represented by the ratio of the numbers divided by total follicle numbers, was equal among the three groups (Table 2). No difference was noted regarding the uterine endometrial thickness and bilateral uterine artery blood perfusion, the pulsatility index, and the resistance index (data not shown) among the three groups of participants.

Across the entire study population, 2.2 ± 1.9 mature oocytes were retrieved, resulting in 1.6 ± 1.6 normal 2-pronuclei pre-embryos; among those embryos, 0.9 ± 1.0 were freshly transferred. Remaining embryos were cryopreserved. There were significantly higher numbers of total and mature oocytes retrieved in Group C. There were significant differences among the three groups in the number of mature oocytes, the total oocytes retrieved, the normal fertilized two-pronuclei pre-embryos, and the good embryos obtained. There were also significant differences among the three groups in the number of embryos transferred (Table 2). The clinical pregnancy rates (15.1%, 20.8%, 33.3%, respectively) and the live birth rates (16.3%, 12.2%, and 26.1%, respectively) did not show significant differences among the three groups (Table 2).

The OSIs of 767.1 ± 579.1 IU observed in this study indicates that women with POR might achieve proper follicle maturation under vaginal administration of rhFSH. The OSI value for those women in Group C was almost half the value of those in the other two groups. In our study, we also observed a FORT level of 86 to 122% and a FOI of 108 to 144%, which indicates the efficacy of the administration mode as well as the dynamic response of ovarian folliculogenesis to exogenous Gns.

### 3.4. Adverse Reactions

No women presented with symptoms and signs of OHSS. Only seven women out of 266 (2.6%) felt a painful sensation during the vaginal administration, while most of the other women did not feel any discomfort. Nonetheless, none of the patients withdrew from the study. No discomfort, bleeding, or painful sensation was noted in most women receiving vaginal administration of rhFSH. In fact, up to 80% of the women were unaware of the injection until after completion of the Gn vaginal administration. Most women expressed satisfaction with the intermittent vaginal administration instead of the daily injections they had experienced in their previous IVF treatments.

## 4. Discussion

In this study, we proved the efficacy of intermittent vaginal administration of Gns in POR women receiving COH in IVF treatment. Controlled ovarian hyperstimulation employing Gn administration for multiple ovarian follicle growth is one of the most effective measures for increasing pregnancy rates in IVF treatments [33,34]. In the past two decades, most gonadotropin preparations used for infertility treatment were recombinant preparations that needed to be injected under the skin nearly every day to be effective, due to relatively short half-life of rhFSH—every 17 to 35 h for follitropin alfa and follitropin beta, respectively [35,36]. In the present study, the novel intermittent vaginal administration protocol was used for COH. Women with POR only received an average of three doses of Gns to complete the COH process. The intermittent vaginal administration protocol could not only decrease the burden of daily injections but also save over half of the overall costs of Gn doses in comparison to the costs of conventional practice for POR women. The dynamic analysis of folliculogenesis represented by lower OSI and higher FORT and FOI indicates an efficient response of POR women to the present Gn administration. The clinical pregnancy rate of 18.1% and the life birth rate of 16.7% further indicate the feasibility of this administration mode.

Studies have indicated that the serum FSH level should be exceeded to enhance preantral and early antral follicles’ progression to maturation [37,38]. The serum FSH level of 5–8 IU/L after 5 days of exogenous rhFSH administration in IVF treatment has been noted to be associated with the growth of follicles [32]. In this study, the serum concentrations of FSH over 5 IU/L in most time points detected at day 5, 8, and 11 (i.e., 3 days after vaginal administration) were investigated, and over 8 IU/L in 80% of all time points investigated indicated a sufficient serum level for follicular growth employing the present intermittent vaginal administration of Gns. Furthermore, a high estradiol level is supposed to exert its negative feedback to suppress the FSH level [39]. However, sustained elevated serum FSH up to days 8 and 11 of the treatments was noted when the serum level of estradiol higher was than 1000 pg/mL (Figure 4B). The smaller plasma elimination rate constant, the slower total body clearance, and the extended half-life of 69.99 h of rhFSH administered vaginally could be used to explain the sustained high serum FSH concentration [18]. The proficiency of vaginal rhFSH administration could be based mostly on these pharmacokinetic characteristics.

The total dose of Gns used (1318.4 ± 634.4 IU) in this study, however, was lower than the suggested dose of 3000 IU rhFSH in conventional daily subcutaneous injections for women with normal ovulation [40,41,42]. Our doses were also much lower than those in the study of Berkkanoglu et al., where they used up to 3749 to 4575 IU rhFSH for COS in POR women [43]. A total dose of 4000 IU Gns was also used in a large randomized controlled trial that included Bologna POR patients [44]. A recent study of POR women employing a combination of early-stop GnRH-agonist and letrozole priming with a multiple-dose GnRH-antagonist cycle showed that the total dose of Gns was 4400 to 6200 IU to complete the COH [45]. Thus, nearly 67 to 75% of the overall cost of the Gn doses could be saved by employing the protocol of intermittent vaginal administration of Gns in this study, in comparison to the conventional practice for POR women. A much-reduced cost of medicine and a decrease in the number of injections needed for COH reduces the emotional stress among infertile women.

The dynamic analysis of the folliculogenesis data indicated an efficient response of POSEIDON group 4 women to the present Gn administration. The OSI of 835.9 ± 588.2 IU, 746.0 ± 608.0 IU, and 397.6 ± 263.9 IU observed, respectively, in women with AMH of 0.16 ± 0.15, 0.73 ± 0.18, and 2.55 ± 1.31 in the present study indicated that follicle maturation might be reached with fewer doses of rhFSH administered vaginally in POR women. This result was very similar to that in our recent study employing the same vaginal administration of rhFSH, in which an OSI of 768.7 ± 532.6 IU was noted in women with AMH of 0.72 ± 0.37 [18], and to the results of a previous study in which OSI of 365.2 ± 321.4 and 616.4 ± 688.5 IU was noted in those women with AMH 2.7–8.5 and 0.25–1.1 ng/mL, respectively, where the women received conventional daily Gn injections for COH [41]. We observed a FORT level of 86 to 122% in the present study, which was higher than the low FORT level of 30% in a hypo-response and the FORT level approaching 80% in an adequate response for conventional COS [30]. Compared to the response of FOI of around 50% for conventional COS [31], the FOI in the present study was 108 to 144%, indicating a much higher response in our subjects and supporting the increased efficacy of the vaginal administration mode.

The clinical pregnancy rate of 18.1% in this study is comparable to, and even better than, the very low pregnancy rate of 1.5 to 10.2% in a recent POR study [46], and to the 12.8% rate for mild ovarian stimulation and the 13.6% rate for conventional COH in a randomized controlled trial of POR [47]. The live birth rate of 16.7% is also comparable to that of 12.92% in the GnRH antagonist cycle in POR women [48], and higher than the low live birth rate of 2.6% in Bologna POR patients undergoing natural cycle IVF [23] and the cumulative live birth rate of 9.68 to 17% after three complete IVF cycles in POSEIDON group 4 women [49,50]. It will be interesting to explore whether Gns administered vaginally will also be transported to the uterine endometrium in a higher concentration, which would then stimulate important local paracrine factors to enhance embryo implantation. A depressed estrogen receptor and a highly elevated progesterone receptor (PR) in uterine endometrium were noted in our previous study of rats using intermittent vaginal rhFSH administration [18]. If the pattern of elevated PR in rats receiving intermittent vaginal rFSH injections also presents in humans, its clinical application needs further investigation.

A majority of injected Gns in this study would be expected to be transported either through the lymphatic system or via venous circulation (Figure 1). A recent study indicated that medicines usually travel through the lymphatic vessels and the lymph nodes before reaching systemic circulation, after subcutaneous administration [51,52]. That study indicated that the bioavailability of medicines in subcutaneous tissue may be due to differences in the proteolytic degradation and the uptake of the injected therapeutic protein (TP) by the lymphatic plexus. TPs with a molecular weight greater than 16 kDa, especially the more than 80% of TPs with a molecular weight greater than 24 kDa, were absorbed via the lymphatic capillaries [51,53]. In the present study, the Gns (rhFSH, 30 kDa) were expected to be absorbed mostly through the lymphatic system, then transported to their target tissue. In women, the lymph vessels run from the upper portion of the vagina toward the uterine cervix, drain into the external iliac nodes and the internal iliac nodes and end in the hypogastric lymph glands [54]. As described above, the lymphatic system of the upper part of the vagina, in direct communication with the uterus, may represent a potential route for direct passage to the uterus of substances applied into the vagina. However, whether Gns administered under the sub-epitheliem and the interstitial space of the vaginal are also transported through the lymphatic system, as has been described for subcutaneous tissue, needs further study. In addition, different types of vaginal aminopeptidase enzymatic activities in humans were noted, which may result in the degradation of protein and peptide drugs in the vagina [55]. Furthermore, the administration of rhFSH through the vaginal mucosa might have effects on the microbial system and lead to changes in some potential molecular functions linked to cell metabolism, motility, genetic information, the immune system, and signaling processes [56], which influence successful reproduction. Future studies will be needed to clarify whether alterations in local microbial systems and vaginal enzyme degradation occur, what percentage of administered Gns will be absorbed and drained through the lymphatic system, and what percentage of administered Gns will be drained through the countercurrent blood circulation.

Regarding the safety of rhFSH for women undergoing IVF, no teratogenic, mutagenic, or clastogenic effects were noted after rhFSH administration in animal models and in a series of trials and meta-analysis in humans [57,58,59]. However, the long-term effect of vaginal administration of Gns should be further inspected via future prospective trials. Most of our subjects did not feel painful sensations throughout the process of vaginal administration, as the innervation of the upper portion of the vagina is predominantly from the autonomic nervous system and the uterovaginal nerve plexus, a subsidiary of the inferior hypogastric plexus. Only the distal 1/5 of the vaginal wall receives somatic innervations, which have sensory innervation. Thus, the administration of rhFSH close to the uterine cervix is crucial, and not only favors transfer to the uterus and the ovaries, but is also painless [60].

Several advantages support drug administration in the vagina, which including the first uterine pass mechanism, high permeability for low-molecular-weight medicines, steady drug levels due to relatively low enzymatic activity in the vagina, and a rich blood supply in the wider vaginal mucosa [61,62]. In recent years, vaginal drug administration has been broadened beyond the treatment of local vaginal infections. Spray-dried microparticles with chitosan glutamate multiunit carriers of zidovudine were developed to facilitate microbicide delivery for antiherpes activity in the human vaginal epithelium [63]. More promising, in the fight against HIV/AIDS, polymeric nanoparticles and freeze-dried bigels have been shown to enhance the distribution and retention of promising antiretroviral compounds in the vaginal mucosa [5,6,64].

In a very recent report, a novel vaginal drug delivery system—a non-injectable route for cloprostenol—was invented to trigger luteolysis and successfully induce sows to farrow [65]. Thus, the vaginal administration of medication may be employed to improve various aspects of female health, including reproductive functions. Future inventions on the Depot formula or microneedle for the vaginal administration of Gns should be considered to improve the efficacy of drug absorption and clinical effects. A therapeutic mode could be invented, similar to the Norplant implant, to facilitate the timely release of Gns to stimulate ovarian follicle growth for the first 8–10 days of the menstrual cycle, followed by a short pause of 1–2 days, then the release of a progesterone component to help the luteal support of embryo implantation. In addition, nanosystems, such as liposomes, nanoparticles, micelles with tunable surface properties, and thermogelling nanocomposites, provide protection and sustained release of loaded drugs and have been exploited to improve local drug delivery, biodistribution, retention, and uptake of peptides and proteins in vulvovaginal tissues [66,67]. Further development will be focused toward the development of nanoparticle-based Gn delivery to the vagina for more efficient COH.

In general, due to inter-individual variability of the vagina’s physiological characteristics, such as pH value, micro-flora, cervical mucus, and cyclic changes during the menstrual cycle, the vagina cavity is still a challenging site for medication administration [68]. There are also some limitations in the present study, including the following: (1) the concentration of rhFSH or Gns in the target tissue of the uterus and the ovaries could not be detected; and (2) the results of the present study were not compared to other administration modes, such as conventional daily subcutaneous injection of Gns. Although there are few limitations on drug administration via the vagina, we believe that vaginal Gn administration is a favorable way for women receiving IVF, as most infertile women need to receive vaginal ultrasound scanning every 3–4 days for the follow-up of follicular growth since the start of the menstrual cycle (Figure 2). Gns can be given to most women through the vaginal route once the ultrasound scanning is completed and the vaginal ultrasound probe is removed. It takes only 1–2 min to complete the vaginal administration of Gns, according to our clinical experience in the treatments of over four hundred women and thousands of vaginal injections.

## 5. Conclusions

In conclusion, the vaginal administration of Gn has been demonstrated to be effective for ovarian hyperstimulation in POSEIDON Group 4 women, which provides a new scenario of COS for women with poor ovarian response. Further and larger prospective studies are needed to prove whether this administration mode can be employed in most women receiving COH in different protocols of COH. Studies on the mechanisms of how vaginally administered gonadotropins are transported to reach their target tissue may also be further investigated. The development of nanoparticle-based Gn delivery to the vagina is also expected.

## Figures and Tables

**Table 1:** Demographic patterns in women of poor ovarian reserve receiving vaginal administration of gonadotropins.

	All	AMH < 0.5 (Group A)	0.5 ≤ AMH < 1.2 (Group B)	AMH ≥ 1.2 (Group C)	F	*p* Value	Post-Hoc
	Mean ± SD	Mean ± SD	Mean ± SD	Mean ± SD			Bonferroni
Demographic patterns							
Age (years)	40.3 ± 4.2	40.8 ± 4.0	39.3 ± 4.2	39.1 ± 5.0	3.836	0.023 *	
BMI (Kg/M^2^)	22.1 ± 3.0	22.0 ± 3.0	22.9 ± 3.0	20.4 ± 2.4	6.388	0.002 *	B > C, A > C
AMH (ng/mL)	0.52 ± 0.84	0.16 ± 0.15	0.72 ± 0.18	2.55 ± 1.31	357.780	<0.001 *	C > B > A
AFC (numbers)	2.8 ± 2.2	2.2 ± 1.6	3.4 ± 2.2	6.0 ± 2.7	49.376	<0.001 *	C > B > A
FSH_day3 (IU/L)	17.905 ± 14.300	21.040 ± 15.759	11.321 ± 5.814	9.175 ± 3.465	16.407	<0.001 *	A > B, A > C
Primary infertility (%)	44.50%	47.60%	39.60%	33.30%		0.276	
Years infertility	5.5 ± 4.6	5.6 ± 4.6	5.9 ± 5.1	4.4 ± 3.4	1.052	0.351	
Previous IVF cycles	1.9 ± 2.4	2.1 ± 2.6	1.4 ± 1.7	1.8 ± 1.7	2.200	0.113	
Causative factors of infertility							
Tubal (%)	8.30%	9.20%	0.00%	18.50%		0.013 *	
Endometriosis/adenomyosis (%)	23.80%	20.00%	34.00%	29.60%		0.082	
Myoma (%)	10.60%	13.00%	7.50%	0.00%		0.089	
More than 38 years (%)	55.50%	61.10%	43.40%	40.70%		0.020 *	
Other ovulatory problem (%)	39.20%	38.90%	39.60%	40.70%		0.982	
Male (%)	5.70%	2.20%	17.00%	7.40%		<0.001 *	
Other Uterine (%)	3.00%	2.20%	5.70%	3.70%		0.413	

Abbreviations: BMI: body mass index; AMH: anti-Müllerian hormone; FSH: follicle-stimulating hormone; AFC: antral follicles at the beginning of menstrual cycle, indicated by the presence of 3–8 mm small follicles at baseline. All data presented as mean ± SD, except categorical variables. Statistical analysis was performed by one-way ANOVA followed by Bonferroni’s post hoc tests. Group A: AMH < 0.5 ng/mL; Group B: 0.5 ng/mL ≤ AMH < 1.2 ng/mL; Group C: AMH ≥ 1.2 ng/mL. * *p* < 0.05.

**Table 2:** Clinical and embryological parameters after vaginal administration of Gns.

	All	AMH < 0.5 (Group A)	0.5 ≤ AMH < 1.2 (Group B)	AMH ≥ 1.2 (Group C)	F	*p* Value	Post Hoc
	Mean ± SD	Mean ± SD	Mean ± SD	Mean ± SD			Bonferroni
Injection number	3.0 ± 0.9	3.0 ± 0.9	3.1 ± 0.7	3.0 ± 0.7	0.495	0.610	
Days of hCG	10.4 ± 2.8	10.2 ± 3.0	10.9 ± 2.2	10.3 ± 2.1	1.380	0.253	
Total dose Gn	1318.4 ± 634.4	1343.1 ± 661.3	1291.9 ± 637.0	1191.6 ± 414.1	0.721	0.487	
AFC (numbers)	2.8 ± 2.2	2.2 ± 1.6	3.4 ± 2.2	6.0 ± 2.7	49.376	<0.001 *	C > B > A
hCGd follicle 9–11/total follicle	0.282 ± 0.257	0.287 ± 0.275	0.265 ± 0.222	0.269 ± 0.185	0.174	0.840	
hCGd follicle 12–15/total follicle	0.327 ± 0.286	0.316 ± 0.310	0.323 ± 0.227	0.406 ± 0.210	1.152	0.318	
hCGd follicle 16–21/total follicle	0.392 ± 0.278	0.397 ± 0.295	0.412 ± 0.233	0.325 ± 0.239	0.933	0.395	
hCGd Endometrium thickness	8.553 ± 2.640	8.346 ± 2.676	9.314 ± 2.544	8.172 ± 2.276	2.836	0.061	
Oocyte_total	3.0 ± 2.3	2.4 ± 1.6	3.63 ± 2.1	6.0 ± 3.6	39.212	<0.001 *	C > B > A
Oocyte_mature	2.2 ± 1.9	1.8 ± 1.4	2.4 ± 1.8	4.2 ± 3.3	23.700	<0.001 *	C > B, C > A
Oocyte_immarture	0.8 ± 0.98	0.6 ± 0.7	1.2 ± 1.2	1.7 ± 1.2	26.976	<0.001 *	C > B > A
2PN	1.6 ± 1.6	1.2 ± 1.3	2.1 ± 1.7	3.1 ± 1.8	21.919	<0.001 *	C > B > A
Good embryos	0.9 ± 1.0	0.7 ± 0.9	1.3 ± 1.3	2.0 ± 1.5	18.420	<0.001 *	C > B > A
Total egg/embryos frozen	0.560 ± 1.067	0.600 ± 0.979	0.434 ± 1.201	0.556 ± 1.368	0.496	0.609	
Number_fresh ET #	0.8 ± 1.0	0.7 ± 0.9	1.1 ± 0.9	1.7 ± 1.2	15.212	<0.001 *	C > B > A
Clinical pregnancy (%) #	18.1%	15.1%	20.8%	33.3%			
Live birth (%) #	16.7%	16.3%	12.2%	26.1%		0.355	
OSI	767.1 ± 579.1	835.9 ± 588.2	746.0 ± 608.0	397.6 ± 263.9	6.707	0.001 *	B > C, A > C
FORT	1.0 ± 0.8	0.9 ± 0.7	1.2 ± 1.1	0.9 ± 0.6	2.957	0.054	
FOI	1.3 ± 1.0	1.3 ± 1.0	1.4 ± 1.2	1.1 ± 0.5	1.038	0.356	

Abbreviations: AMH: anti-Müllerian hormone; hCG: human chorionic gonadotropin; Gn: gonadotropin; AFC: antral follicle counts; hCGd: day of human chorionic gonadotropin injection; 2 PN: two pronuclei; ET: embryo transfer; OSI: ovarian sensitivity index, the dose of rhFSH used divided by the number of mature oocytes obtained; FORT: follicular output rate, the ratio of preovulatory follicle count (14–22 mm in diameter) on hCG day 100/small antral follicle count (3–8 mm in diameter) at baseline; FOI: follicle to oocyte index, the ratio between the number of oocytes obtained and the number of antral follicles at the beginning of COS. All data presented as mean ± SD, except categorical variables. Statistical analysis was performed by one-way ANOVA followed by Bonferroni’s post hoc tests. Group A: AMH < 0.5 ng/mL; Group B: 0.5 ng/mL ≤ AMH < 1.2 ng/mL; Group C: AMH ≥ 1.2 ng/mL. * *p*< 0.05. #: indicates per oocyte retrieval.

## Data Availability

The data that support the findings of this study are available from Chao Chin Hsu. Restrictions apply to the availability of these data, which were used under license for this study. The data presented in this study are available on request from Chao Chin Hsu with the permission of the Taiwan United Birth-promoting Experts Fertility Clinic.
